# *NmrB* (*AN9181*) expression is activated under oxidative stress conditions acting as a metabolic repressor of *Aspergillus nidulans*

**DOI:** 10.3389/fmicb.2024.1373469

**Published:** 2024-04-18

**Authors:** João M. P. Jorge, Celso Martins, Patrícia Domingos, Tiago M. Martins, Diego O. Hartmann, Gustavo H. Goldman, Cristina Silva Pereira

**Affiliations:** ^1^Instituto de Tecnologia Química e Biológica António Xavier, NOVA University Lisbon, Av. da República, Oeiras, Portugal; ^2^Faculdade de Ciências Farmacêuticas de Ribeirão Preto, Universidade de São Paulo, Ribeirão Preto, Brazil

**Keywords:** organic chemicals, stress responses, transcriptomics, aspergilli, phenylcoumaran benzylic ether reductase-like domain (IPR045312), NmrA-like domain (IPR008030), global regulators, oxidative stress

## Abstract

Aspergilli comprise a diversity of species that have been extensively studied due to their catabolic diversity, biotechnological and ecological value, and pathogenicity. An impressive level of structural and functional conservation has been shown for aspergilli, regardless of many (yet) cryptic genomic elements. We have hypothesized the existence of conserved genes responsive to stress in aspergilli. To test the hypothesis of such conserved stress regulators in aspergilli, a straightforward computational strategy integrating well-established bioinformatic tools was used as the starting point. Specifically, five transcriptome-based datasets on exposure to organic compounds were used, covering three distinct *Aspergillus* species. Among the identified up-regulated genes, only one gene showed the same response in all conditions, *AN9181*. This gene encodes a protein containing a phenylcoumaran benzylic ether reductase-*like* domain and a Nitrogen metabolite repressor regulator domain (NmrA). Deletion of this gene caused significant phenotypic alterations compared to that of the parental strain across diverse conditions. Specifically, the deletion of *AN9181* raised the mutant’s metabolic activity in different nitrogen sources. The acquired data supports that *AN9181* acts by repressing (slowing down) *A. nidulans* growth when exposed to aromatic compounds in a concentration dependent manner. The same phenotype was observed for amphotericin B. Finally, *AN9181* underwent differential upregulation under oxidative stress conditions. Collectively, the data suggest that *AN9181*, herein assigned as *NmrB* (Nitrogen Metabolite Repression Regulator B), builds up the genetic machinery of perception of oxidative stress by negatively regulating growth under such conditions.

## 1 Introduction

The aspergilli comprise a diverse group of saprophytic filamentous fungi covering over 200 million years of evolution (Galagan et al., 2005; Gupta et al., 2021) that are found virtually everywhere. Aspergilli have been extensively studied mostly due to high biotechnological value, for example *Aspergillus oryzae* and *A. niger* are used for the production of sake and citric acid, respectively (Machida et al., 2008; Behera, 2020), and *A. terreus* was the first known producer of lovastatin, a cholesterol-lowering statin (Hajjaj et al., 2001; Pearce, 2019). Aspergilli are also important human pathogens, mainly *A. fumigatus*, which is the major causative agent of aspergillosis (Steenwyk et al., 2020). Recently, this species was included in the list of priority fungal pathogens published by the World Health Organization (World Health Organization, 2022). Despite the extensive knowledge already acquired about aspergilli genomes, many gene functions remain unknown or poorly understood (Galagan et al., 2005).

Fungi show great potential to degrade and/or mineralize various aromatic compounds, including environmental pollutants, a capacity mostly attributed to their impressive catabolic capabilities (Harms et al., 2011; Varela et al., 2015, 2017; Martins et al., 2019). Regardless of efficient degradation, a great stress is imposed; this has been linked to increased pathogenic potential of a fungal community (Martins et al., 2018) and to the production of virulent airborne spores from aspergilli strains in environments polluted with aromatic compounds (Martins et al., 2023). Earlier studies on the genome’s structure and evolution in aspergilli revealed an impressive level of structural and functional conservation and synteny (Galagan et al., 2005; Steenwyk et al., 2020), both in coding and non-coding regions, regardless of evident evolutionary rearrangements. Taking the catabolism of aromatic compounds as an example, in the different available Dikarya genomes, the pathway gene clusters are found often in an array with the peripheral pathway genes (Martins et al., 2019). Such high level of genomic similarity in aspergilli, underlines the hypothesis of the existence of conserved regulatory genes responsive to variable chemical stresses (Galagan et al., 2005; Martins et al., 2019). In the present study, we integrated the transcriptome signatures of different aspergilli (three species) to distinct organic compounds, gathering original and publicly available datasets. To identify genes showing the same response across the different datasets, a straightforward computational strategy that allows comparing transcriptomic-based datasets initially collected in distinct *Aspergillus* species was used. Only one gene – *AN9181*, assigned as *NmrB* (Nitrogen Metabolite Repression Regulator B), showed the same response in all the datasets. The phenotype of *A. nidulans* single deletion-mutant ∆*AN9181* was compared to that of the wild type, covering for example germination fitness, growth rate and susceptibility to distinct chemical stressors. The collected data suggest that *NmrB* negatively regulates the metabolism of *A. nidulans* in specific stress conditions.

## 2 Materials and methods

### 2.1 Chemicals

Bromoquinol (BMQ) was purchased from Alfa Aesar; 6-iodoquinoline (IDQ) and resveratrol (RVT) from TCI Europe; hydrogen peroxide (H_2_O_2_) from Merck; dimethyl sulfoxide from Fisher Chemical, and the remaining compounds from Sigma Aldrich, namely pentachlorophenol (PCP), triclosan (TCS), salicylate (Sal), benzo[a]pyrene (BaP), congo red, sodium benzoate, menadione sodium bisulfite (MSB), amphotericin B (Amph B), caspofungin (CSP), itraconazole (ITZ), miconazole (Mic) and 2,7-dichlorofluorescin diacetate (DCFH-DA).

### 2.2 Transcriptomics analysis of *Aspergillus fumigatus* upon exposure to iodoquinoline

*Aspergillus fumigatus* CEA17 reference strain was propagated at 37°C in solid complete medium [1% D-glucose, 0.2% Peptone, 0.1% Yeast extract, 0.1% Casamino acids, 50 mL of a 20× salt solution, 0.1% trace elements, 0.1% Vitamin solution, 2% agar, pH 6.5 with NaOH]. The composition of the trace elements, vitamins, and nitrate salts has been described previously (Kafer, 1977).

The Minimal Inhibitory Concentration (MIC) for fungal growth inhibition of IDQ, initially acquired from The Pathogen Box (www.pathogenbox.org/), was defined following the standard methodology implemented by the Clinical and Laboratory Standards Institute (CLSI, 2018). The compound’s antifungal activity was analyzed by serial dilutions using MIC assay (0 to 25 μM) in MOPS buffered RPMI 1640 medium (Sigma-Aldrich), pH 7.0 in 96-wells plates. In each well, a total of 1×10^4^ conidia of *A. fumigatus* wild-type strain was inoculated. Plates were incubated at 37°C without shaking for 48 h. Non-inoculated controls were done in parallel. All experiments were done in triplicate.

The preparation of total RNA samples for RNA-seq Expression Profiling was as follows. Erlenmeyer flasks (125 mL) were used to inoculate 1×10^7^ spores in 30 mL of Vogel’s Minimal Media (Vogel, 1956) and incubated for 16 h at 37°C, 180 rpm. The medium was then exchanged, and 0, 0.5x MIC (=0.35 μM) or 2x MIC (=1.4 μM) of IDQ was added and incubated for 4 h at 37°C, 180 rpm. Six replicates for each condition were prepared. At the end of the incubation period, the cultures were filtered and frozen immediately in liquid nitrogen. Total RNA from six mycelia per condition were extracted using RNeasy Plant Mini Kit (Qiagen), according to the manufacturer’s protocol, a TissueLyser LT (Qiagen) for cell disruption, and approximately 30 mg of poly(vinylpolypyrrolidone) per sample. RNA quality (integrity) was evaluated using a Nucleic Acid QC - Fragment Analyzer.

For single-end RNA sequencing (RNA-seq), libraries were generated using the Smart-Seq2® mRNA assay (Illumina, Inc.) according to the manufacturer’s instructions. Six samples were indexed and sequenced on the Illumina NextSeq550 (20 M reads per sample). Generated FastQ files were analyzed with FastQC, and any low-quality reads were trimmed with Trimmomatic (20). All libraries were aligned to the corresponding model fungus *A. fumigatus* A1163 genome assembly (ASM15014v1) with gene annotations from Ensembl Fungi v. 45 using HISAT2 v. 2.1.0 (Kim et al., 2015), and only matches with the best score were reported for each read. All RNA-seq experiments were carried out in three biological replicates. Differential expression analysis was performed using DESeq2 v. 1.24.0 (Love et al., 2014). The genes that showed more than log_2_ 1-fold expression changes with *p-adj value* < 0.05 were considered as significantly differentially expressed (IDQ dataset in Supplementary Dataset 1).

### 2.3 Selection of transcriptomics datasets

The transcriptomics datasets were selected based on the following stringent rules: only studies on aspergilli were considered (i), comprising an exposure period lower than six days (ii) to an organic compound displaying broad environmental, biotechnological or health relevance (iii) with a molar mass below 500 g·mol^−1^. Consequently, we selected five transcriptome-based datasets: the catabolism of the simple aromatic hydrocarbon salicylate (Sal) in *A. nidulans* (Martins et al., 2021); the mode of action of the polyphenol resveratrol (RVT) in *A. flavus* (Wang et al., 2015); the degradation of the polycyclic aromatic hydrocarbon benzo[a]pyrene (BaP) in *Aspergillus* sp. (Loss et al., 2019); the inhibitory effects of the quinoline bromoquinol (BMQ) (Ben Yaakov et al., 2017) and of 6-iodoquinoline (IDQ) in *A. fumigatus* (see above). The full gene lists (up-regulated only) used in our analysis are available in Supplementary Dataset 2.

### 2.4 Comprehensive co-expression analysis

**Figure 1:**
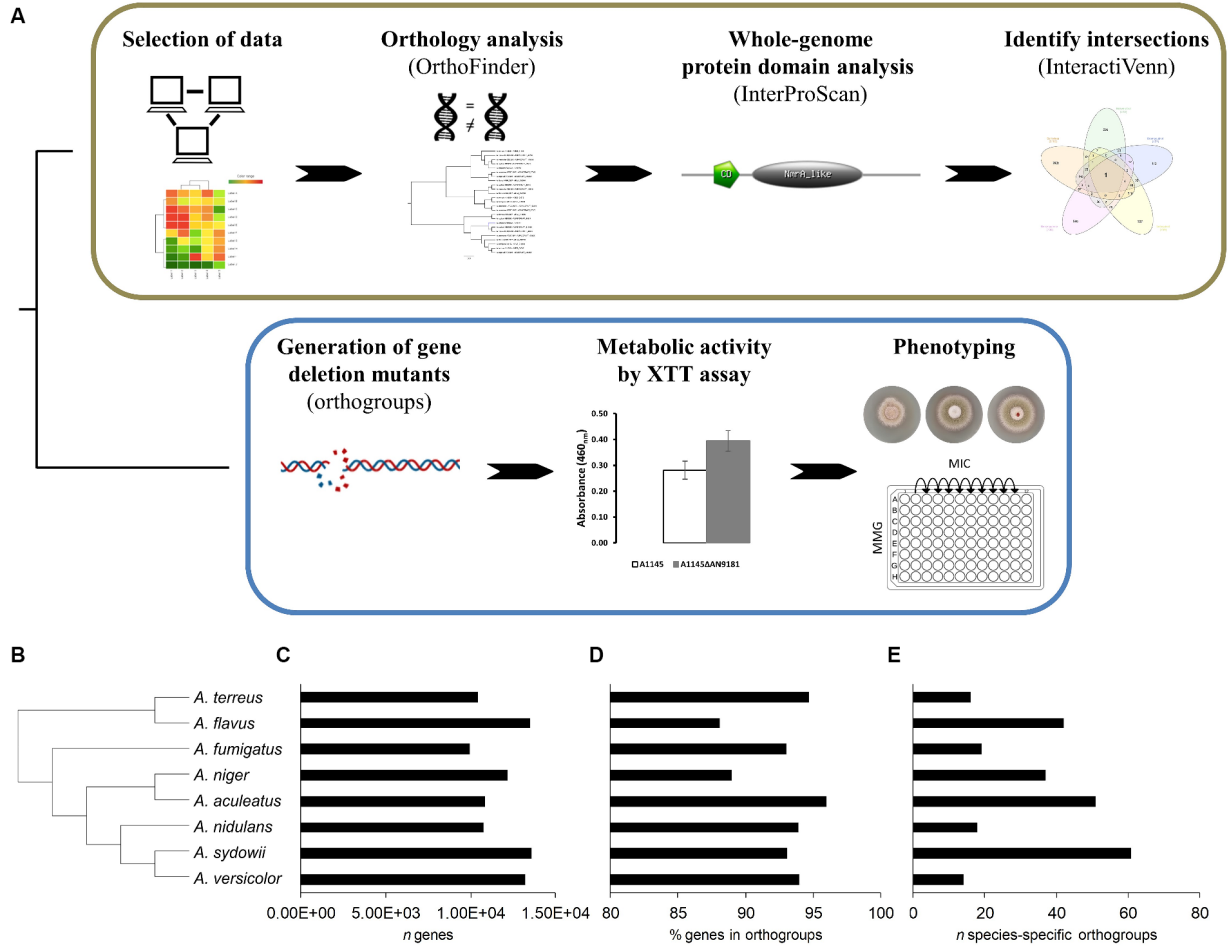
Schematic overview of the COmpregensive CO-expression Analysis (COCOA) strategy developed in this work. **(A)** – the iterative use of the well-established bioinformatics tools OrthoFinder, InterProScan and InteractiVenn upon selection of suitable and meaningful transcriptome-based datasets is displayed. Laboratory assays implemented to understand the biological relevance of the gene identified by COCOA. Results of the orthology analysis performed using the genomes of eight *Aspergillus* spp. **(B)** – the species tree, **(C)** – the number of genes of each analyzed genome, **(D)** the percentage of genes in orthogroups and **(E)** the number (*n*) of species-specific orthogroups are depicted.

The COCOA strategy uses a set of established bioinformatics tools (Figure 1A) as detailed below. First, we performed the curation and validation of the five selected datasets by reprocessing the raw data to obtain the gene counts using the HISAT2 methodology (Kim et al., 2015) and identifying the differentially expressed genes using the *DESeq2* R-based package (Love et al., 2014). Then, a full protein-translated genome orthology transformation to *A. nidulans* (the “receiver species”) was performed for eight distinct aspergilli available in the FungiDB database: *A. aculeatus* ATCC16872, *A. flavus* NRRL3357, *A. fumigatus* A1163, *A. nidulans* FGSCA4, *A. niger* ATCC13496, *A. sydowii* CBS593.65, *A. terreus* NIH2624, and *A. versicolor* CBS583.65 (Stajich et al., 2012). For the orthology analyzes, we used OrthoFinder (Emms and Kelly, 2015, 2017, 2019) because of its proven excellent performance compared to other orthology tools (Emms and Kelly, 2015). The use of these additional genomes than those strictly necessary adds robustness to the construction of gene trees, resulting in better discrimination and stringency levels between orthogroups (Emms and Kelly, 2015, 2017, 2019; Martins et al., 2020). We transposed the list of the up-regulated genes in the five datasets to the corresponding *A. nidulans* orthologues. After that, the co-expressed genes were identified using the online tool InteractiVenn (Heberle et al., 2015). Finally, we performed a whole-genome protein domain analysis for the protein-translated genome of the “receiver species,” aiming to obtain hints on the putative functions played by the co-expressed genes, necessary when a functional annotation is lacking.

### 2.5 *Aspergillus nidulans* culture media

If not mentioned, assays used minimal medium with glucose (10 g·L^−1^) and nitrate (MMGN), as follows: thiamine (0.01 g·L^−1^), 5% (v/v) nitrate salts solution [NaNO_3_ (120.0 g·L^−1^), KCl (10.4 g·L^−1^), MgSO_4_·7H2O (10.4 g·L^−1^) and KH_2_PO_4_ (30.4 g·L^−1^)], 0.1% (v/v) trace elements solution [ZnSO_4_·7H2O (22.0 g·L^−1^); H_3_BO_3_ (11.0 g·L^−1^); MnCl_2_·4H2O (5.0 g·L^−1^); FeSO_4_·7H2O (5.0 g·L^−1^); CoCl_2_·6H_2_O (1.7 g·L^−1^); CuSO_4_·5H_2_O (1.6 g·L^−1^); Na_2_MoO_4_·2H_2_O (1.5 g·L^−1^), and Na_4_EDTA (50.0 g·L^−1^)], and the pH was adjusted to 6.5 with NaOH. Whenever needed, the media was jellified with 1.5% agar (solid cultivation). The MMG is similar to that described above except that sodium nitrate was removed. Most functional assays used MMG supplemented or not with a defined amount of a N source as mentioned in the results section. Moreover, in all comparative assays of mutant versus parental strain, the medium was supplemented with the essential nutrients: uracil (1.12 g·L^−1^), uridine (1.22 g·L^−1^), riboflavin (2.5 mg·L^−1^) and pyridoxine (0.05 mg·L^−1^).

### 2.6 Half maximal effective concentration (EC_50_)

EC_50_ levels of each organic compound were assessed on basis of hyphal radial growth rate for the strain *A. nidulans* FGSC A4 grown in petri dishes (55 mm) containing the MMGN (jellified) and supplemented with either BaP (1.0–3.5 mM); BMQ (0.1–1.0 mM); Sal (75–400 mM); IDQ (0.002–1.0 mM); RVT (0.005–1.5 mM); or sodium benzoate (25–200 mM). Controls without the organic compounds were also made. The assay was carried out by inoculating 2×10^5^ conidia into the center of the plate, and incubate at 30°C for 120 h. Radial growth of mycelia (colony diameter in cm) was measured using a Vernier caliper (error ± 0.05 mm). The plates that did not displayed visible growth were visualized under microscope to confirm growth inhibition. The EC_50_ values were calculated using the XLSTAT tool (Microsoft Excel) with the Gompertz model.

### 2.7 *Aspergillus nidulans* submerged cultures

For the targeted gene expression analysis of the two genes of the AN9181 orthogroup, *Aspergillus nidulans* (5×10^5^ spores·mL^−1^) was pre-grown during 24 h in 12-well plates (2 mL of MMGN per well) at 30°C with gentle agitation (100 rpm), in the dark. After the pre-growth phase, the selected organic compounds were added to the cultures at a defined concentration (Time 0). The concentrations used were as follows: 0.2 mM for BaP [similar to the concentration used by Loss et al. (2019)]; 0.5 mM for BMQ (half of the minimal inhibitory concentration described by Ben Yaakov et al. (2017); 0.3 mM for PCP (concentration previously tested before by Varela et al. (2017), and 0.2 mM for TCS to match the range used for the other organic compounds. For the gene expression analysis of *AN9181* in the presence of H_2_O_2_, A1145 strain (1×10^6^ spores·mL^−1^) was pre-grown during 140 h in 12-well plates (2 mL of MMG per well) at 30°C with gentle agitation (100 rpm), in the dark. After the pre-growth phase, H_2_O_2_ was added to the cells at a defined concentration (1.0 –4.0 mM) and incubated at 30°C for 30 min. Controls without addition of H_2_O_2_ were grown as well. Mycelia samples were collected from the 12-well plates, and immediately frozen using liquid nitrogen until further analysis.

### 2.8 Real-time quantitative polymerase chain reaction

Total RNA extraction and cDNA synthesis were performed using the RNeasy Plant Mini Kit (Qiagen) and the iScript cDNA Synthesis Kit (Bio-Rad), and the RT-*q*PCR performed as previously reported (Martins et al., 2020). The oligonucleotide pairs for specific *A. nidulans* genes (Supplementary Table 1) were designed using the Primer-Blast web tool (www.ncbi.nlm.nih.gov/tools/primer-blast/), and purchased from STAB Vida Lda. (Portugal). The RT-*q*PCR analysis was performed in a CFX96 Thermal Cycler (Bio-Rad), using the SsoFast EvaGreen Supermix (Bio-Rad), 250 nM of each oligonucleotide and the cDNA template equivalent to 10 ng of total RNA, at a final volume of 10 μL per well, in at least three biological replicates. The PCR conditions were as follows: enzyme activation at 95°C for 30 s; 40 cycles of denaturation at 95°C for 5 s, and annealing/extension at 60°C for 15 s; and the melting curve obtained from 65 to 95°C, consisting of 0.5°C increments for 5 s. Data were analyzed with the CFX Manager software (Bio-Rad). In more detail, the expression of each gene was taken as the relative expression compared to the time zero (before incubation with the tested compounds) or as the relative expression compared to the no addition of the stressor. The expression of all target genes was normalized by the expression of the histone *H2B* gene (*AN3469*) (Supplementary Dataset 3), used as the internal control. Statistical analyzes used the XL-STAT (Addinsoft) software, and multiple Student’s *t-tests*. Differences in gene expression with a *p-value* below 0.05 were considered statistically significant.

### 2.9 Generation of single-gene deletion mutants

The gene *AN9181* was replaced with *Aspergillus fumigatus pyrG* gene (*pyrG*^Afu^) in *Aspergillus nidulans* A1145 and A1149, both auxotrophic strains (*pyrG*^−^). Deletion cassettes combining the 5′ and 3′-flanking regions of each target gene with *pyrG*^Afu^ were obtained by fusion PCR and were used to transform *A. nidulans* A1145 or A1149 protoplasts and plated onto selective media to generate single-deletion mutant strains (Supplementary Table 2). Isolated transformants were cultivated on the selective media for three generations to assure stable mutations. In greater detail, the deletion cassettes were constructed using a fusion PCR protocol. Six primers (P1-P6) were designed, based on sequences from the *Aspergillus* Genome Database (www.aspgd.org), and analyzed using the NetPrimer web tool (www.premierbiosoft.com/NetPrimer/AnalyzePrimer.jsp) (listed in Supplementary Table 3). PCR reactions were performed in a T100 Thermal Cycler (Bio-Rad; conditions in Supplementary Table 4). *A. fumigatus pyrG* was amplified from plasmid pCDS60 (FGSC, Kansas City, MO, United States) using primers CDS164 and CDS165. Flanking fragments upstream and downstream of the gene were amplified with primer pairs P1/P3 and P4/P6, respectively, using genomic DNA from *A. nidulans* A4 as template. The final cassette was produced by fusing the flanking regions with the *A. fumigatus pyrG* using nested primers P2 and P5 for the target gene. PCR products were cleaned with NZYGelPure kit (NZYTech). Then, to produce transformable protoplasts, *A. nidulans* mycelia grown overnight from 10^8^ conidia in 50 mL MMGN with the appropriate nutritional supplements (30°C, 90 rpm), recovered by centrifugation and washed (0.6 M MgSO_4_), were digested in 20 mL enzymatic mix (300 mg lysing enzymes from *Trichoderma harzianum*, 150 μL β-glucuronidase from bovine liver, type B-1, and 150 mg Driselase from *Basidiomycetes* sp., all from Sigma-Aldrich) in osmotic medium (1.2 M MgSO_4_·7H_2_O and 10 mM sodium phosphate buffer pH 6.5; final pH adjusted to 5.8 with Na_2_HPO_4_) for 20 h at 30°C, 90 rpm. The protoplasts suspension was overlaid (2:1) with trapping buffer (0.6 M sorbitol and 100 mM Tris–HCl pH 7.0), and then recovered by centrifugation (1,500 *g*, 4°C, 15 min, swing-bucket rotor), washed three times with 10 mL of ST10 buffer (1.2 M sorbitol and 10 mM Tris–HCl pH 7.5) and finally resuspended in 1 mL of the same buffer and incubated overnight at 4°C. In the next day, the protoplasts were collected by centrifugation (1,000 *g*, at 4°C, 2 min), and resuspended in 700 μL cold STC buffer (1.2 M sorbitol, 10 mM Tris–HCl pH 7.5 and 10 mM CaCl_2_). The obtained protoplasts (100 μL) were then transformed by mixing with the cleaned fusion PCR product (10 μL), subsequently adding freshly filtered polyethylene glycol (PEG) solution (25% (w/v) in STC buffer; 50 μL) and kept in an ice bath for 25 min. Then, additional PEG solution was added (1 mL), gently mixed using a micropipette and placed at room temperature for 25 min. For the single mutants A1145*ΔAN9181* and A1149*ΔAN9181*, 100 μL of the transformation mix were plated onto a selective medium containing glucose (5.0 g·L^−1^), yeast extract (5.0 g·L^−1^), sucrose (342.3 g·L^−1^), riboflavin (2.5 mg·L^−1^), pyridoxin (0.05 mg·L^−1^), 0.1% (v/v) trace elements solution (see above), and 1.5% agar. The selective plates were incubated for 3 to 4 days at 30°C. All transformants were morphologically identical to the parental strain. Two isolated A1145Δ*AN9181* transformants (randomly selected) were streaked onto complete medium plates containing glucose (10.0 g·L^−1^), peptone (2.0 g·L^−1^), yeast extract (1.0 g·L^−1^), casein hydrolysate (1.0 g·L^−1^), 5% (v/v) nitrate salts solution (see above), 0.1% (v/v) trace elements solution, riboflavin (2.5 mg·L^−1^), pyridoxin (0.05 mg·L^−1^), pH 6.5, and incubated at 30°C for 4 days. Three generations of each transformant were grown to assure stable mutations and then grown again in MMG without uracil and uridine to confirm the prototrophy to the compounds of the mutant strains (Supplementary Figure 1).

### 2.10 Confirmation of gene replacement

DNA from each transformant was extracted with Quick-DNA™ Fungal/Bacterial Microprep Kit (Zymo Research), and diagnostic PCR was performed with primers P1 and P6 for each gene. Based on amplicon size (3,222 bp for WT and 3,904 bp for A1145Δ*AN9181*, Supplementary Figure 2A), it was possible to confirm the correct gene replacement of the transformants. To obtain further confirmation, the PCR products were digested for 1 h with the restriction enzymes ApaI or KpnI selected to give differential digestion patterns for mutant and wild-type strains. Supplementary Figure 2B show the products of diagnostic PCR and restriction enzymes digestion for the A1145Δ*AN9181* selected strain.

### 2.11 Cellular metabolic activity measured by XTT assay

Cell viability was evaluated using the XTT assay in 96-well plates (200 μL per well) using Malt Extract medium or MMG supplemented with 10 mM N source, and an inoculum of 1×10^6^ spores·mL^−1^, and incubated at 30°C for 24 h (triplicates). After, 10 μL of a solution containing 4.6 mg·mL^−1^ of XTT and 0.104 mg·mL^−1^ of menadione was added to each well, incubated for further 2 h, and the absorbance measured (460 nm).

### 2.12 *Aspergillus nidulans* growth and inhibition assays

The strains’ spore germination fitness, *i.e*., number of spores that are able to form a colony, was evaluated by spreading 100 spores onto solid media, then counting the colony forming units (CFUs) daily, during five days (triplicates). The radial growth diameter of each strain (inoculum: 1 μL of a suspension of 2×10^8^ spores·mL^−1^) in solid MMG, supplemented or not with specific compounds (*viz.* congo red, H_2_O_2_, MSB, Amph B, CSP and ITZ) was measured after five days of incubation (30°C, dark, triplicates). The MIC of each antifungal was determined using the micro-broth dilution method (MMG, 96-well plates, 200 μL per well), testing specific concentration ranges for Amph B (250–550 mg·L^−1^), CSP (60–120 mg·L^−1^) and ITZ (0.2–0.5 mg·L^−1^). An inoculum of 1×10^6^ spores·mL^−1^ was used, and the plates were incubated at 30°C for 48 h. Negative controls were done in parallel. The lowest concentration that showed no growth under microscopic observation was considered the MIC.

### 2.13 ROS quantification

Intracellular ROS was quantified using DCFH-DA. Cultures were grown in MMG (140 h, 30°C); then 2.5 μg·mL^−1^ of DCFH-DA was added and incubated (30°C, 30 min). H_2_O_2_ was added to the cells at increasing concentrations, from 1 mM to 4 mM, and the incubation step repeated. The cell suspension was disrupted in a TissueLyzer LT (Qiagen) with a metal bead at a maximum speed (3 cycles of 1 min). The fluorescence intensity of the supernatant (recovered by centrifugation: 12000 *g*, 10 min) was measured using a Tecan Infinite M Nano^+^ Microplate (Männedorf, Switzerland) as follows: excitation length 485/9; emission 528/20; optics, top; read speed, normal; gain, 89; number of flashes, 25; integration time, 40 μs. The fluorescence intensity (per mycelia dry weight) was normalized against the control (no H_2_O_2_ added).

## 3 Results and discussion

### 3.1 Genes comprised in the AN9181 orthogroup underwent differentially upregulation during growth in the presence of aromatic compounds

The orthology analysis (Figure 1A) revealed a total of 11,992 orthogroups among the eight analyzed *Aspergillus* spp. genomes (Supplementary Dataset 4). Among these, only 258 were species-specific orthogroups, comprising 0.7% of the total number of genes. Figure 1B displays the phylogenetic relations computed by the orthology analysis, and Figures 1C–E provide for each species the total number of genes, the percentage of genes in orthogroups and the number of species-specific orthogroups, respectively. The number of species-specific orthogroups (Figure 1E) is not necessarily correlated with the percentage of genes in orthogroups (Figure 1D) nor the genome size (Figure 1C). For instance, *A. versicolor* possesses the third larger genome among the eight analyzed *Aspergillus* spp. yet displays one of the highest percentages of genes present in orthogroups but the lowest number of species-specific orthogroups. On the other hand, *A. sydowii*, which possesses a genome size comparable to that of *A. versicolor* and only a slightly lower percentage of genes belonging to orthogroups, is the species presenting the higher number of species-specific orthogroups. This is an indication of a higher occurrence of gene duplication events (Emms and Kelly, 2015) in *A. sydowii* compared to other aspergilli, a feature also visible in *A. aculeatus* and *A. flavus*. Finally, *A. fumigatus* and *A. nidulans* present similarly sized genomes, as well as a comparable percentage of genes included in orthogroups and number of species-specific orthogroups.

**Figure 2:**
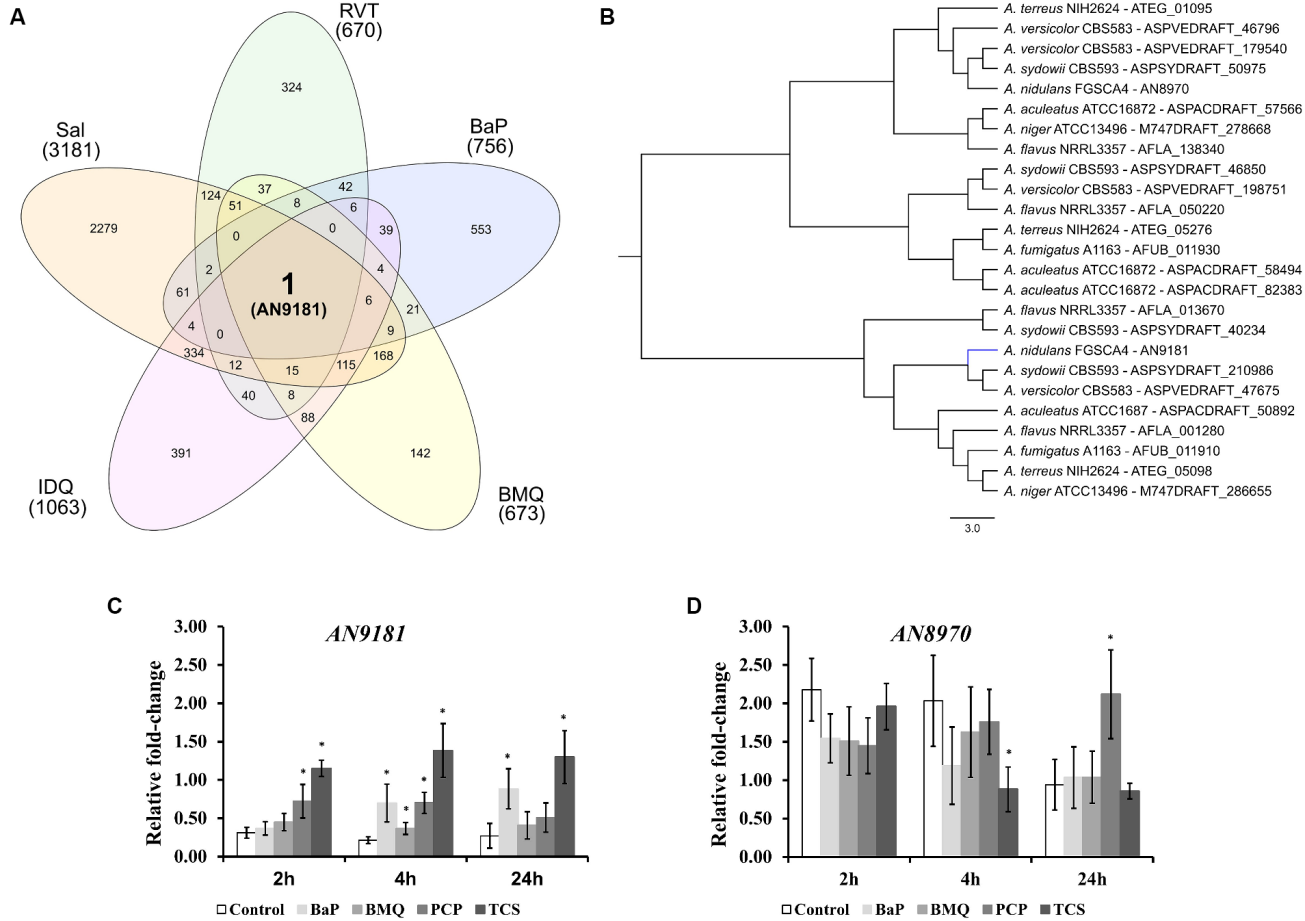
Different expressed genes in the five transcriptomic datasets. **(A)** – upon orthology transformation of the transcriptome-based datasets to the “receiver species” *Aspergillus nidulans*, *AN9181* was the only gene found consistently up-regulated in all the analyzed datasets. BaP (Benzo[a]pyrene); BMQ (Bromoquinoline); Sal (Sodium Salicylate); IDQ (6-iodoquinoline) and RVT (Resveratrol). **(B)** – the AN9181 orthogroup comprises 25 genes present in the genomes of the eight orthology-analyzed *Aspergillus* species, which are separated into two distinct clusters in the gene-tree obtained upon OrthoFinder analysis. **(C,D)** – targeted gene expression analysis (assessed by RT-*q*PCR) is displayed for AN9181 **(C)** and AN8970 **(D)**.

Upon transposing the differentially expressed genes (up-regulated) from the five transcriptomic datasets analyzed to the corresponding orthologues in the genome of *A. nidulans* (Supplementary Dataset 2) we analyzed the set of genes present in at least three, four or five transcriptome-based datasets on exposure to selected organic compounds. We observed that, out of the 241 genes present in at least three datasets, several are predicted to be transporters or secondary metabolism-related genes (Supplementary Dataset 5). Twenty-two genes are present in at least four datasets (Table 1), of which a single gene, *AN9181*, is present in all five datasets (Figure 2A). This gene is part of an orthogroup that contains two genes in *A. nidulans*, being the other one *AN8970*.

**Table 1:** List of genes present in at least four distinct transcriptome-based datasets out of the five analyzed using the COCOA strategy.

Gene ID	Product description	InterPro code	InterPro domain
AN0016	Putative nonribosomal peptide synthase	IPR000873 IPR001242 IPR006162 IPR009081 IPR010071 IPR020806 IPR020845 IPR023213 IPR036736 IPR042099 IPR045851	AMP-dependent synthetase/ligase domain; Condensation domain; Phosphopantetheine attachment site; Phosphopantetheine binding ACP domain; Amino acid adenylation domain; Polyketide synthase, phosphopantetheine-binding domain; AMP-binding, conserved site; Chloramphenicol acetyltransferase-like domain superfamily; ACP-like superfamily; ANL, N-terminal domain; AMP-binding enzyme; C-terminal domain superfamily
AN0029	Putative transmembrane transporter	IPR011701 IPR020846 IPR036259	Major facilitator superfamily; Major facilitator superfamily domain; MFS transporter superfamily
AN2959	Has domain(s) with a predicted role in transmembrane transport and integral component of membrane localization	IPR011701 IPR020846 IPR036259	Major facilitator superfamily; Major facilitator superfamily domain; MFS transporter superfamily
AN3225	Putative cytochrome P450	IPR002403 IPR001128 IPR036396 IPR017972	Cytochrome P450, E-class, group IV; Cytochrome P450; Cytochrome P450 superfamily; Cytochrome P450, conserved site
AN4643	Putative cytochrome P450	IPR001128 IPR002401 IPR036396 IPR017972	Cytochrome P450; Cytochrome P450, E-class, group I; Cytochrome P450 superfamily; Cytochrome P450, conserved site
AN5310	Has domain(s) with predicted FAD binding, oxidoreductase activity and role in metabolic process	IPR002938 IPR036188	FAD-binding domain; FAD/NAD(P)-binding domain superfamily
AN5553	Putative cytochrome P450	IPR001128 IPR002401 IPR036396 IPR017972	Cytochrome P450; Cytochrome P450, E-class, group I; Cytochrome P450 superfamily; Cytochrome P450, conserved site
AN6450	Tetrahydroxynaphthalene reductase	IPR002347 IPR020904 IPR036291	Short-chain dehydrogenase/reductase SDR; Short-chain dehydrogenase/reductase, conserved site; NAD(P)-binding domain superfamily
AN7154	protein of unknown function	IPR008030 IPR036291	NmrA-like domain; NAD(P)-binding domain superfamily
AN7359	Putative cytochrome P450	IPR001128 IPR002401 IPR036396 IPR017972	Cytochrome P450; Cytochrome P450, E-class, group I; Cytochrome P450 superfamily; Cytochrome P450, conserved site
AN7772	Putative cytochrome P450	IPR001128 IPR002401 IPR017972 IPR036396	Cytochrome P450; Cytochrome P450, E-class, group I; Cytochrome P450, conserved site; Cytochrome P450 superfamily
AN7969	Putative cytochrome P450	IPR001128 IPR002401 IPR036396 IPR017972	Cytochrome P450; Cytochrome P450, E-class, group I; Cytochrome P450 superfamily; Cytochrome P450, conserved site
AN7972	Has domain(s) with a predicted role in transmembrane transport and integral component of membrane localization	IPR011701 IPR020846 IPR036259	Major facilitator superfamily; Major facilitator superfamily domain; MFS transporter superfamily
AN8354	Has domain(s) with predicted NAD binding, oxidoreductase activity, acting on the aldehyde or oxo group of donors, NAD or NADP as acceptor activity and role in cellular amino acid metabolic process, oxidation–reduction process	IPR008030 IPR036291 IPR045312	NmrA-like domain; NAD(P)-binding domain superfamily; Phenylcoumaran benzylic ether reductase-like
AN8952	Putative cytochrome P450	IPR001128 IPR002401 IPR036396	Cytochrome P450; Cytochrome P450, E-class, group I; Cytochrome P450 superfamily
AN8970	Ortholog of *A. nidulans* FGSC A4: AN9181	IPR016040 IPR036291	NAD(P)-binding domain; NAD(P)-binding domain superfamily
AN9005	Putative polyketide synthase (PKS)	IPR001227 IPR009081 IPR011032 IPR013217 IPR013968 IPR014030 IPR014031 IPR014043 IPR016035 IPR016036 IPR016039 IPR018201 IPR020807 IPR020841 IPR020843 IPR029063 IPR032821 IPR036291 IPR036736 IPR042104 IPR049551 IPR049552	Acyl transferase domain superfamily; Phosphopantetheine binding ACP domain; GroES-like superfamily; Methyltransferase type 12; Polyketide synthase, ketoreductase domain; Beta-ketoacyl synthase, N-terminal; Beta-ketoacyl synthase, C-terminal; Acyl transferase; Acyl transferase/acyl hydrolase/lysophospholipase; Malonyl-CoA ACP transacylase, ACP-binding; Thiolase-like,Beta-ketoacyl synthase, active site; Polyketide synthase, dehydratase domain; Polyketide synthase, beta-ketoacyl synthase domain; Polyketide synthase, enoylreductase domain; S-adenosyl-L-methionine-dependent methyltransferase superfamily; Polyketide synthase, C-terminal extension; NAD(P)-binding domain superfamily; ACP-like superfamily; Polyketide synthase, dehydratase domain superfamily; Polyketide synthase, dehydratase domain, C-terminal; Polyketide synthase, dehydratase domain, N-terminal
AN9044	Has domain(s) with predicted FMN binding, catalytic activity, oxidoreductase activity and role in oxidation–reduction process	IPR001155 IPR013785 IPR044152	NADH:flavin oxidoreductase/NADH oxidase, N-terminal; Aldolase-type TIM barrel; NADPH dehydrogenase YqjM-like
AN9161	Has domain(s) with predicted FAD binding, oxidoreductase activity and role in metabolic process	IPR002938 IPR036188	FAD-binding domain; FAD/NAD(P)-binding domain superfamily
AN9181	Ortholog of *A. nidulans* FGSC A4: AN8970	IPR008030 IPR036291 IPR045312	NmrA-like domain; NAD(P)-binding domain superfamily; Phenylcoumaran benzylic ether reductase-like
AN10259	Putative cytochrome P450	IPR001128 IPR002401 IPR036396 IPR017972	Cytochrome P450; Cytochrome P450, E-class, group I; Cytochrome P450 superfamily; Cytochrome P450, conserved site
AN11681	protein of unknown function	No domain	No domain

The genes’ IDs and Product Descriptions based on FungiDB annotation are available. The assigned InterPro codes and names of the predicted domains upon InterProScan analysis are also provided.

The AN9181 orthogroup (gene tree displayed in Figure 2B) comprises 25 genes across the eight analyzed aspergilli genomes, namely four in *A. aculeatus*, four in *A. flavus*, two in *A. fumigatus*, two in *A. nidulans* (as mentioned), two in *A. niger*, four in *A. sydowii*, three in *A. terreus*, and four in *A. versicolor*. The existence of the AN9181 orthogroup is therefore highly conserved in these aspergilli. The presence of four genes belonging to the AN9181 orthogroup in *A*. *sydowii*, *A. aculeatus* and *A. flavus* reinforces the indication of higher occurrence of gene duplication events in these species. This is an indication that the AN9181 orthogroup, like many other regulatory genes, is undergoing rapid evolution in *Aspergillus* sp. as a response to environmental changes and adaptive lifestyles. This plasticity is often associated with the evolution/transition to pathogenic lifestyles in aspergilli (Rokas, 2022). The 25 genes that compose the AN9181 orthogroup are largely uncharacterized with neither relevant information in FungiDB and nor predicted protein interactions. However, the orthologous gene *cip1* of *Candida albicans* is involved in oxidative stress response, being regulated by the transcription factor Cap1p (Wang et al., 2006; Znaidi et al., 2009). This past evidence raises the hypothesis that *AN9181* participates in stress responses.

Through a computational protein domain analysis (InterProScan), we observed that the *AN9181* encodes a protein containing a phenylcoumaran benzylic ether reductase-*like* domain (IPR045312) and a Nitrogen metabolite repressor regulator (NmrA)*-like* domain (IPR008030), while *AN8970* encodes a protein containing a NAD(P)-binding domain (IPR016040). A deletion mutant of *padA*, that encodes a protein also carrying a NmrA-*like* domain of *Dictyostelium discoideum*, showed to be more sensitive to ammonia than the wild-type (Núñez-Corcuera et al., 2008). Moreover, disruption of this gene resulted in phenotypic defects in development and growth, namely, the thermosensitive mutant allele *padA*^−^ showed poor and null growth at permissive and restrictive temperatures, respectively. Deletion of the *NmrA* gene in *A. flavus* reduced growth in several nitrogen (N) sources, but increased conidia and sclerotia production (Han et al., 2016). This mutant strain produces less aflatoxin when cultivated in glutamine and alanine supplemented media, and shows reduced virulence and increased sensitivity in response to rapamycin and methyl methanesulfonate, but not in response to the osmotic stressors NaCl and sorbitol (Han et al., 2016).

The genes *AN9181* and *AN8970* are in separated clusters within the AN9181 orthogroup gene tree (Figure 2B). This observation is consistent with the fact that they possess distinct functional domains (Gabaldón and Koonin, 2013), and therefore, they are not paralogs. Though *AN9181* was the initial candidate, we also evaluated the gene expression of *AN8970* (same orthogroup) aiming to understand which one would be functionally relevant to regulate stress responses. Therefore, the expression profiles of either gene composing the AN9181 orthogroup (*AN9181* and *AN8970*) were evaluated after 2, 4 and 24 h of exposure to four selected organic compounds in a medium containing a poor N source. The organic compounds comprise two which were found in the selected transcriptome datasets: benzo[a]pyrene (BaP), and bromoquinol (BMQ), and two additional aromatic halogenated based compounds, namely pentachlorophenol (PCP) and triclosan (TCS), classified as persistent organic pollutant and contaminant of emergent concern, respectively (Varela et al., 2017). The last two compounds were tested due to their frequent association to soil and water contamination (Czaplicka, 2004; Morgan et al., 2015), and past studies showing that either compound increased the production of virulent aspergilli conidia within soil colonizing fungi (Martins et al., 2023). The *AN9181* revealed lower expression levels compared to *AN8970* (Figures 2C,D). However, compared to control conditions, a significant increase in the expression levels of *AN9181* was systematically noticed after 4 h of exposure to all the tested organic compounds, as well as in additional time-points, namely after 2 h to PCP, 2 and 24 h to TCS, and 24 h to BaP (Figure 2C). Recently, *AN9181* was also found to be up-regulated after fungal growth in a nitrate minimal medium supplemented with either cadmium chloride, congo red or amphotericin B (Antal et al., 2020). In contrast to *AN9181*, the expression profiles of *AN8970* were similar between the control and upon exposure to organic compounds, except for PCP at 24 h and for TCS 4 h (Figure 2D). At the experimental conditions used, the gene expression analysis did not support the idea of a concerted action of the two genes of the AN9181 orthogroup in *A. nidulans*. Based on these results, we focused the remaining analyzes on the *AN9181*.

### 3.2 *AN9181* does not affect germination and growth in solid media but influences metabolic activity in a N source dependent manner

**Figure 3:**
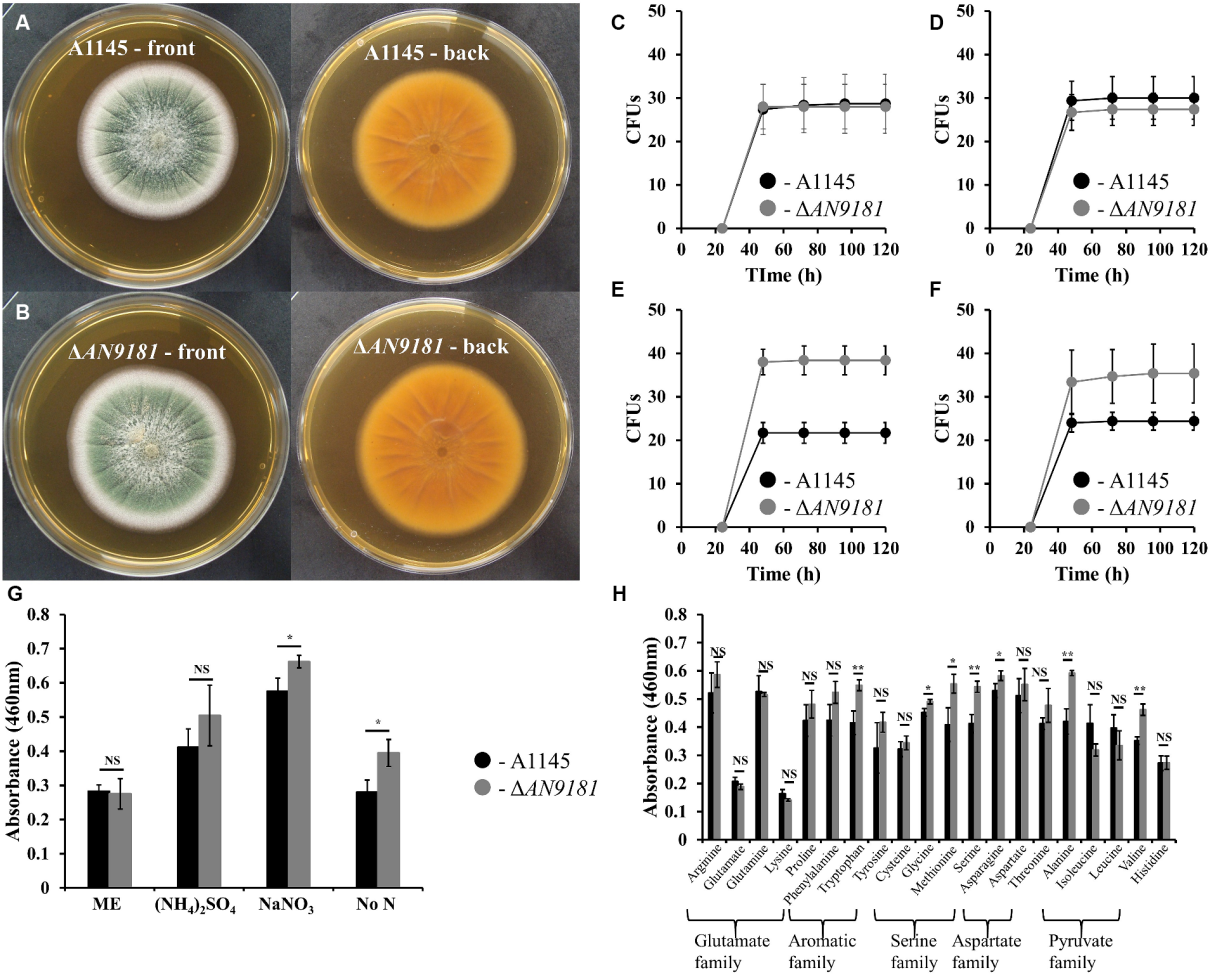
Phenotype features of A1145 and Δ*AN9181*. **(A,B)** – the morphology and pigmentation of A1145 strain **(A)** and Δ*AN9181* strain **(B)** after cultivation at 30°C for five days in Malt Extract Agar. **(C–F)** – colony forming units of A1145 (black) and Δ*AN9181* (gray) strains plated on Malt Extract Agar **(C)**; minimal medium glucose supplemented with 10 mM nitrogen from different sources (NH_4_)_2_SO_4_ **(D)**; 10 mM NaNO_3_ **(E)**; no nitrogen added **(F)**. **(G,H)** – cell viability and proliferation measured by XTT assay. A1145 (black) and Δ*AN9181* (gray) strains were grown in Malt Extract or in minimal medium glucose supplemented with 10 mM nitrogen from the different nitrogen sources **(G)** including amino acids **(H)**. Values and error bars represent the mean and the standard deviation of triplicates. Significant differences (Student’s *t-test*) are marked with asterisks [*). * *p* ≤ 0.05; ** *p* ≤ 0.01, and *** *p* ≤ 0.001. “NS” means non-significant.

To better understand the functional roles of *AN9181*, this gene was deleted and functionally analyzed in *A. nidulans*. The colony morphology of the parental strain and the A1145Δ*AN9181* mutant (hereafter referred to as Δ*AN9181*) on Malt extract agar (MEA) medium were similar (Figures 3A,B). After five days of growth, the colony diameters of either strain in MMG containing a high amount of a non-preferred (*i.e*., poor) N source were also comparable (*i.e*., 71 mM sodium nitrate, Supplementary Figure 3). The conidia viability of either strain (*i.e*., germination fitness) was measured directly by counting the numbers of CFUs. CFUs were similar for both strains when germinated in the rich medium MEA (Figure 3C) and MMG supplemented with either a superior (*i.e*., rich) N source (10 mM of ammonium sulfate) (Figure 3D) or a non-preferred N source at high concentration (71 mM of sodium nitrate) (Supplementary Figure 4A). However, the Δ*AN9181* conidial viability in MMG supplemented with 10 mM sodium nitrate or no added N source were 1.7-fold and 1.5-fold higher, respectively, compared to the parental strain (Figures 3E,F). This result suggests that *AN9181* strongly influences the fitness of conidia germinating in medium having low availability of poor nitrogen sources.

The *AN9181* encodes for a protein containing a NmrA-*like* domain. NmrA is a negative transcriptional regulator of several fungi, involved in the post-translational modulation of the GATA transcription factor AreA (Hensel et al., 1998). In *A. nidulans*, *areA* regulates the activation of genes involved in the utilization of a broad range of N sources: in the presence of rich N sources, e.g., ammonium and glutamine, NmrA binds to AreA preventing nitrogen catabolic gene expression; contrarily in the presence of nitrate, NmrA and AreA dissociation occurs, hence genes involved in the utilization of alternative nitrogen sources are activated (Han et al., 2016). Based on this, we questioned if *AN9181* influences the utilization of different N sources. Specifically, we measured the cellular metabolic activity of both the Δ*AN9181* and the parental strain in distinct media. Measurements were similar in both strains grown in media having rich N sources (*i.e*., MEA and ammonium sulfate) (Figure 3G) and a high availability of sodium nitrate (poor alternative N source) (Supplementary Figure 4B). However, the mutant showed a significant increase in metabolic activity when grown in media with a low availability of a poor N source (10 mM sodium nitrate) or no added N source (Figure 3G). Another mutant - #2Δ*AN9181*, randomly selected, was used for validation purposes. This mutant showed consistently similar metabolic activity in media having rich N sources and increased metabolic activity in media with low availability of a poor N source or no added N source (Supplementary Figure 5). In addition, the Δ*AN9181* and the parental strain were tested in MMG supplemented with 10 mM N of each of the 20 proteinogenic amino acids. Compared to the parental strain, the mutant strain showed higher metabolic activity in tryptophan (aromatic); serine, glycine and methionine (serine family); asparagine (aspartate family); and alanine and valine (pyruvate family) (Figure 3H). Collectively the results suggest that *AN9181* influences the utilization of N sources (hence also spore germination fitness) in a nitrogen type specific manner. Overall, the mutant phenotype suggests that *AN9181* participates in the regulation of nitrogen catabolism in *A. nidulans*, resembling *NmrA* negative regulation of N utilization in many rich N sources. Further assays are however needed to better understand *AN9181* regulatory network in the context of nitrogen utilization.

### 3.3 *AN9181* regulates *Aspergillus nidulans* growth in medium supplemented with sodium salicylate and resveratrol

**Figure 4:**
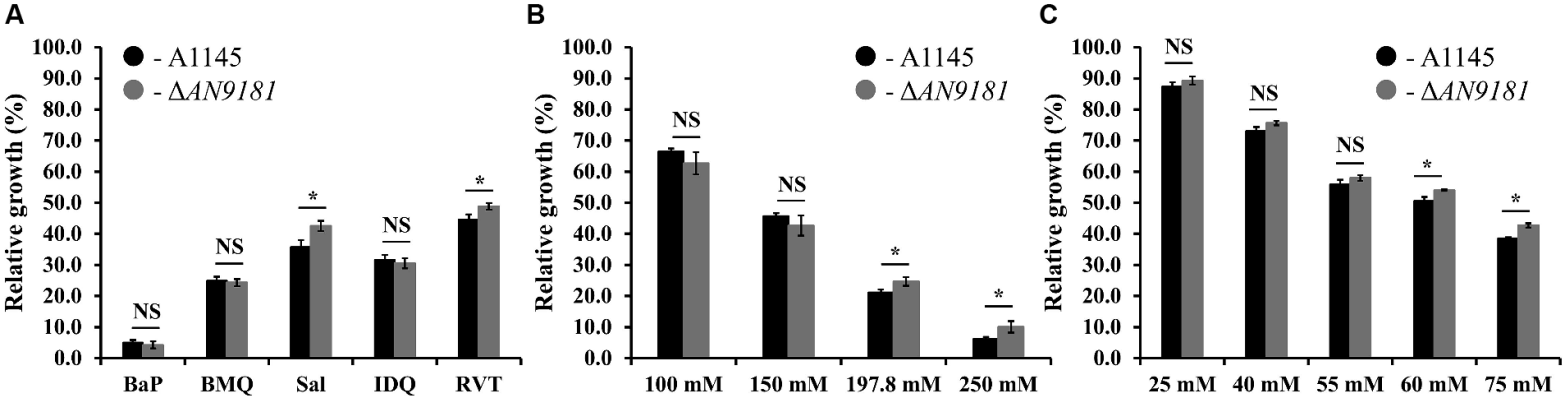
Phenotype features of A1145 and Δ*AN9181*. **(A–C)** relative growth of the A1145 (black) and Δ*AN9181* (gray) strains grown in minimal medium glucose (solid) supplemented with the EC_50_ concentrations of each organic compound compared to the control condition (no chemical addition). BaP (Benzo[a]pyrene, 1.61 mM); BMQ (5-Bromoquinoline, 0.2 mM); Sal (Sodium Salicylate, 197.8 mM); IDQ (6-iodoquinoline, 0.2 mM) and RVT (Resveratrol, 0.45 mM) **(A)**; and with increasing concentrations of sodium salicylate **(B)** or sodium benzoate **(C)**. Values and error bars represent the mean and the standard deviation of triplicates. Significant differences (Student’s *t-test*) are marked with asterisks [*). *p ≤ 0.05; **p ≤ 0.01, and ***p ≤ 0.001. “NS” means non-significant.

The growth of Δ*AN9181* strain in the presence of each organic compound initially covered in the investigated transcriptome datasets was tested. To standardize conditions, all growth assays used MMG supplemented with the compounds under test at their determined EC_50_ values (Supplementary Table 5). In the presence of BaP, BMQ and IDQ no differences in growth were observed between the parental and the mutant strain. On the contrary, in media supplemented with sodium salicylate or resveratrol, the Δ*AN9181* strain grows more than the parental strain (Figure 4A). The cultivation conditions varied from those of the initial studies, including medium composition, time and temperature, as well as the concentration of the aromatic compounds. To test if the latter was influencing the phenotype, we tested the mutant’s growth in media supplemented with increasing concentrations of two selected aromatic compounds. Salicylate is degraded via the catechol branch of the 3-oxoadipate pathway in *A. nidulans* (Martins et al., 2015). The Ascomycota *Phomopsis liquidambari* degrades resveratrol into 3,5-dihydroxybenzaldehyde and 4-hydroxybenzaldehyde, which are subsequently oxidized to 3,5-dihydroxybenzoic acid and 4-hydroxybenzoic acid, respectively (Abo-Kadoum et al., 2022). The latter, is an intermediate of the protocatechuate branch of the 3-oxoadipate pathway used by *A. nidulans* to degrade benzoate. Therefore, it is possible that resveratrol and benzoate pathways are interconnected in *A. nidulans*, thus the phenotype was tested in increasing concentrations of sodium salicylate and sodium benzoate (the last instead of resveratrol). The results showed that the Δ*AN9181* strain growth-phenotype is indeed concentration dependent for sodium salicylate and for sodium benzoate: a clear phenotype is noticed for concentrations >150 mM and > 55 mM, respectively (*i.e*., concentrations equal or above the EC_50_ determined for these compounds; see Supplementary Table 5) (Figures 4B,C).

The absence of a phenotype in three (out of five) of the organic compounds herein tested (Figure 4A) is likely related to the fact that the used concentrations were below the threshold to cause major stress effect under the utilized cultivation conditions. Moreover, the response is also influenced by the regulation of nitrogen catabolism as analyzed above. The observation that the growth phenotype of the Δ*AN9181* strain is sodium salicylate and sodium benzoate concentration dependent, obvious for concentrations above the corresponding EC_50_ values, is consistent with the working hypothesis that *AN9181* regulates stress responsive metabolism.

### 3.4 Δ*AN9181* strain shows decreased susceptibility to amphotericin B, congo red, hydrogen peroxide, and menadione sodium bisulfite

**Figure 5:**
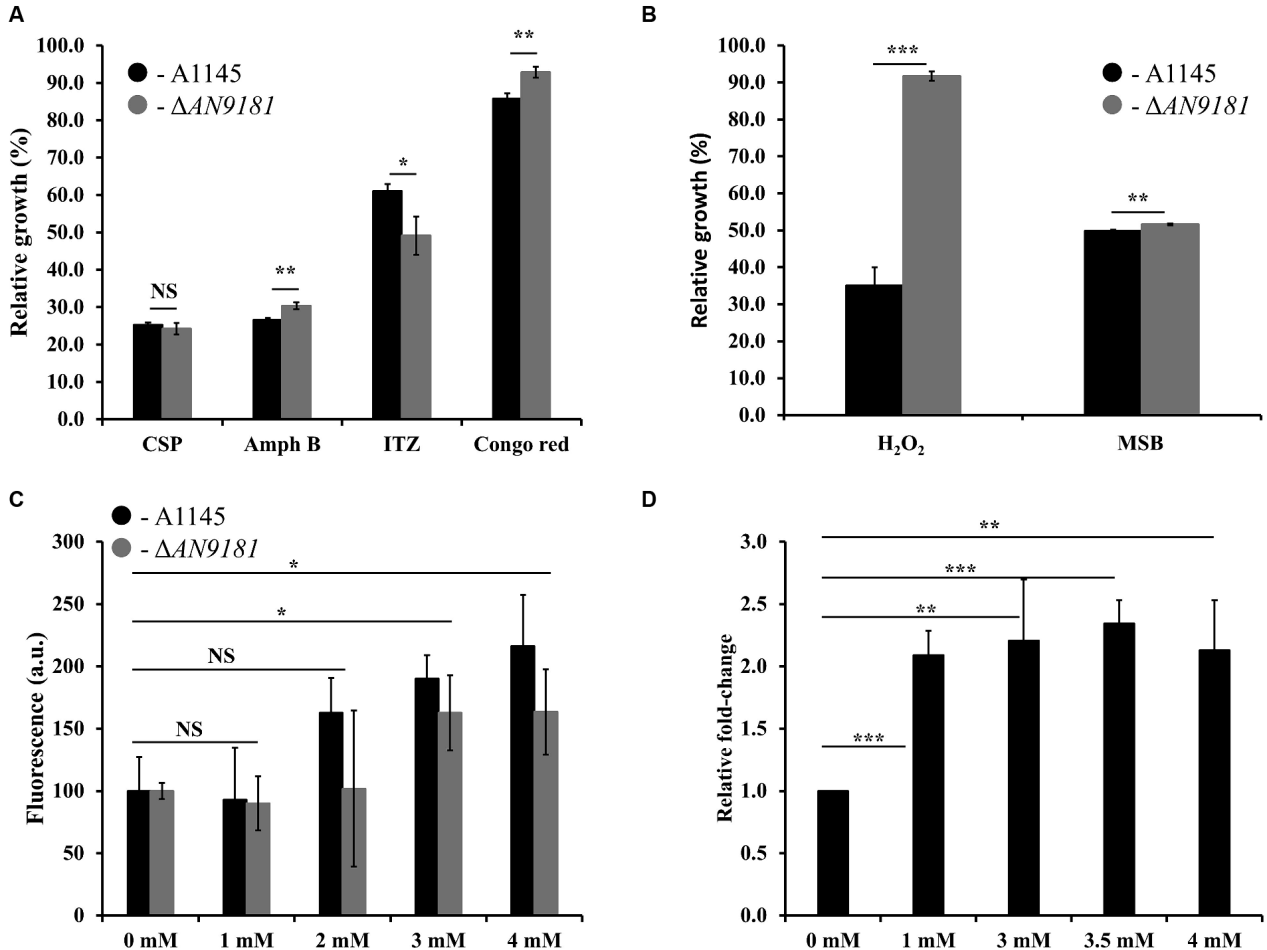
Phenotype features of A1145 and Δ*AN9181*. **(A,B)** relative growth of the A1145 (black) and Δ*AN9181* (gray) strains grown in minimal medium glucose (solid) supplemented with Caspofungin (0.5 mg·L^−1^), Amphotericin B (150 mg·L^−1^), Itraconazole (0.01 mg·L^−1^), Congo Red (0.01 mM), hydrogen peroxide (3.5 mM) and menadione sodium bisulfite (0.1 mM) compared to the control condition (no chemical addition). Values and error bars represent the mean and the standard deviation of triplicates. **(C)** Intracellular ROS production, as indicated by DCFH-DA, quantified for the A1145 (black) and Δ*AN9181* (gray) strains in the presence of different concentrations of H_2_O_2_. The fluorescence intensity per biomass dry weight amount of the control was defined as 100. Values and error bars represent the mean and the standard deviation of at least five replicates. **(D)** targeted gene expression analysis (assessed by RT-*q*PCR) is displayed for AN9181. Fold change relative to the control (without H_2_O_2_ addition) and normalized to *AN3469*. Significant differences (Student’s *t-test*) are marked with asterisks [*). *p ≤ 0.05; **p ≤ 0.01, and ***p ≤ 0.001. “NS” means non-significant.

Recently, it was reported that *AN9181* underwent up-regulation during *A. nidulans* exposure to congo red or amphotericin B grown in a nitrate minimal medium (Antal et al., 2020). This result expands the regulatory impact of *AN9181* under growth in stress conditions. We evaluated if *AN9181* deletion influences the susceptibility to congo red and to different antifungal drugs: amphotericin B (targets the cell membrane), caspofungin (targets the cell wall), and itraconazole (inhibits the synthesis of ergosterol, altering the cell membrane permeability). The results showed that Δ*AN9181* strain susceptibility to amphotericin B is lower compared to the parental strain (Figure 5A). On the contrary, compared to the parental strain, the susceptibility of the mutant strain was similar and higher to caspofungin and itraconazole, respectively (Figure 5A). Similar results were attained with the A1149Δ*AN9181* compared to its corresponding parental strain (Supplementary Figure 6A), notwithstanding that the magnitude of the susceptibility decrease to amphotericin B was more obvious. The determined MICs of each antifungal for the Δ*AN9181* mutant strain and its parental strain validate these results (Table 2). Specifically, the MIC of amphotericin B for the Δ*AN9181* mutant strain increase 1.4-fold compared to that of the parental strain (Table 2). The determined MICs of itraconazole and caspofungin were similar for the mutant and the parental strains (liquid media), regardless of the mutant’s higher susceptibility to itraconazole (solid media). Differences in the growth phenotypes between liquid and solid media have been reported before (Nichols et al., 2011; Martins et al., 2015). These differences can be related to changes in the drug bioavailability and also carbon availability. We also reassessed the growth phenotype of the strains in the presence of congo red (Figure 5A). A significant decrease in the susceptibility of Δ*AN9181* mutant strain compared to the parental strain was noticed. The MIC for congo red in either strain is higher than 256 mg·L^−1^, regardless that the upper inhibitory limit could not be precisely determined due to the strong red color of the media. All tests were conducted in the same growth media, hence the observed differential responses to the antifungal compounds cannot be simply explained by the regulation of nitrogen utilization by *AN9181*.

**Table 2:** MICs of antifungals A1145 and Δ*AN9181* strains.

MIC (mg·L^−1^)			
	Caspofungin	Amphotericin B	Itraconazole
A1145	75	350	0.5
Δ*AN9181*	75	500	0.5

Additionally, the growth phenotype of the strains in the presence of H_2_O_2_ and in the presence of the superoxide menadione sodium bisulfite was assessed. The susceptibility of the Δ*AN9181* mutant strain compared to the parental strain showed a major and minor decrease in the presence of H_2_O_2_ and MSB, respectively (Figure 5B). Using another mutant – #2ΔAN9181 – it was confirmed that the mutant susceptibility to H_2_O_2_ was indeed lower than that of the parental strain (Supplementary Figure 6B).

To complement this result, we evaluated the intracellular ROS levels of the parental and the Δ*AN9181* mutant strains after exposure to specific compounds. The ROS intracellular levels increased upon exposure of either strain to 3 mM or higher concentrations of H_2_O_2_ (Figure 5C). ROS levels increased when the parental strain was exposed to either Amph B or RVT at a concentration where its growth was lower than that of the mutant but slight decreased when exposed to either BMQ and ITZ at concentrations where its growth was similar or higher than that of the mutant, respectively (Supplementary Figure 7). Finally, we observed that the expression of *AN9181* in the parental strain underwent a 2-fold increase in the presence of H_2_O_2_ (≥ 1 mM) compared to the negative control; a clear indication that the *AN9181* is upregulated under oxidative stress (Figure 5D). Collectively the results suggest that *AN9181* participates indeed in stress responses upon exposure to different chemical agents leading to oxidative stress.

## 4 Conclusion

In this study, we aimed to disclose aspergilli genes showing a conserved response when exposed to different organic compounds. For that, we used mostly peer-reviewed public data in combination with a straightforward computational strategy to test the strength of this scientific hypothesis. Relevant transcriptome-based datasets (Wang et al., 2015; Ben Yaakov et al., 2017; Loss et al., 2019; Martins et al., 2021) (one of which was yet unpublished), initially focusing very different biological questions were selected (five datasets in total). The computational strategy herein used allowed to pinpoint *AN9181* as the only gene that showed the same differential upregulation in all five datasets. The AN9181 orthogroup comprises only two genes in *A. nidulans*: *AN9181* and *AN8970*. These genes are located in different clusters of the AN9181 orthogroup gene tree and possess distinct functional domains. Only *AN9181* showed differential upregulation when the fungus was grown in media containing distinct chemical stressors, including two that were used in the previous studies, and two additional toxic aromatic compounds. Therefore, subsequent experimental analyzes focused on the gene *AN9181,* specifically by studying the phenotype of the generated single-deletion mutant compared to the parental strain. This included measuring the viability of the conidia (*i.e*., germination fitness), the metabolic profile in different nitrogen sources, the radial growth in the presence of different stress conditions and the minimal inhibitory concentration to different antifungal drugs. The acquired data showed that the deletion of the gene *AN9181* lead to higher metabolic activities in different N sources, and a decreased susceptibility to sodium salicylate, resveratrol, H_2_O_2_, MSB, congo red and amphotericin B (a clinically relevant antifungal drug). These opening results support the hypothesis that *AN9181* is involved in the regulation against different stress responses, including oxidative stress, in aspergilli, impacting also nitrogen utilization. This gene, *AN9181*, herein assigned as *NmrB* (Nitrogen Metabolite Repression Regulator B), deserves focused in-deep analysis, especially as it is not only conserved in aspergilli but also in several other fungi (*e*. *g*. *Candida* spp., *Fusarium* spp., or *Neurospora* spp., to name a few). The identification of the *AN9181* as a putative regulator of stress in aspergilli could not be anticipated, regardless that some of the transcriptome-based datasets used here were generated years ago. This strategy can help to identify (yet) cryptic genetic elements by reusing publicly available peer-reviewed transcriptome-based data from a diversity of scientific fields. It remains unresolved the nature of its regulation but *AN9181* apparently is able to slow down growth and metabolic activity in conditions that would be otherwise harmful for the fungus. The absence of orthologs in the human genome (according to OrthoMCL) increases the significance of this inaugural observations, especially in the context of invasive aspergillosis.

## Data availability statement

The data presented in the study are deposited in the NCBI repository, accession number PRJNA1065793.

## Author contributions

JJ: Formal analysis, Investigation, Methodology, Writing – original draft, Writing – review & editing. CM: Conceptualization, Investigation, Methodology, Writing – original draft, Writing – review & editing. PD: Formal analysis, Investigation, Writing – review & editing. TM: Formal analysis, Investigation, Writing – review & editing. DH: Investigation, Writing – review & editing. GG: Funding acquisition, Resources, Writing – review & editing. CS: Conceptualization, Funding acquisition, Project administration, Resources, Supervision, Writing – review & editing.
