# Supplementary material for: NmrB (AN9181) expression is activated under oxidative stress conditions acting as a metabolic repressor of Aspergillus nidulans
Source: Front Microbiol. 2024 Apr 18;15:1373469. doi: 10.3389/fmicb.2024.1373469 (PMC11063244; doi:10.3389/fmicb.2024.1373469)
Supplement: Supplementary file 1 [file Data_Sheet_1.docx]

Supplementary Material

# Supplementary Figures and Tables

## Supplementary Figures


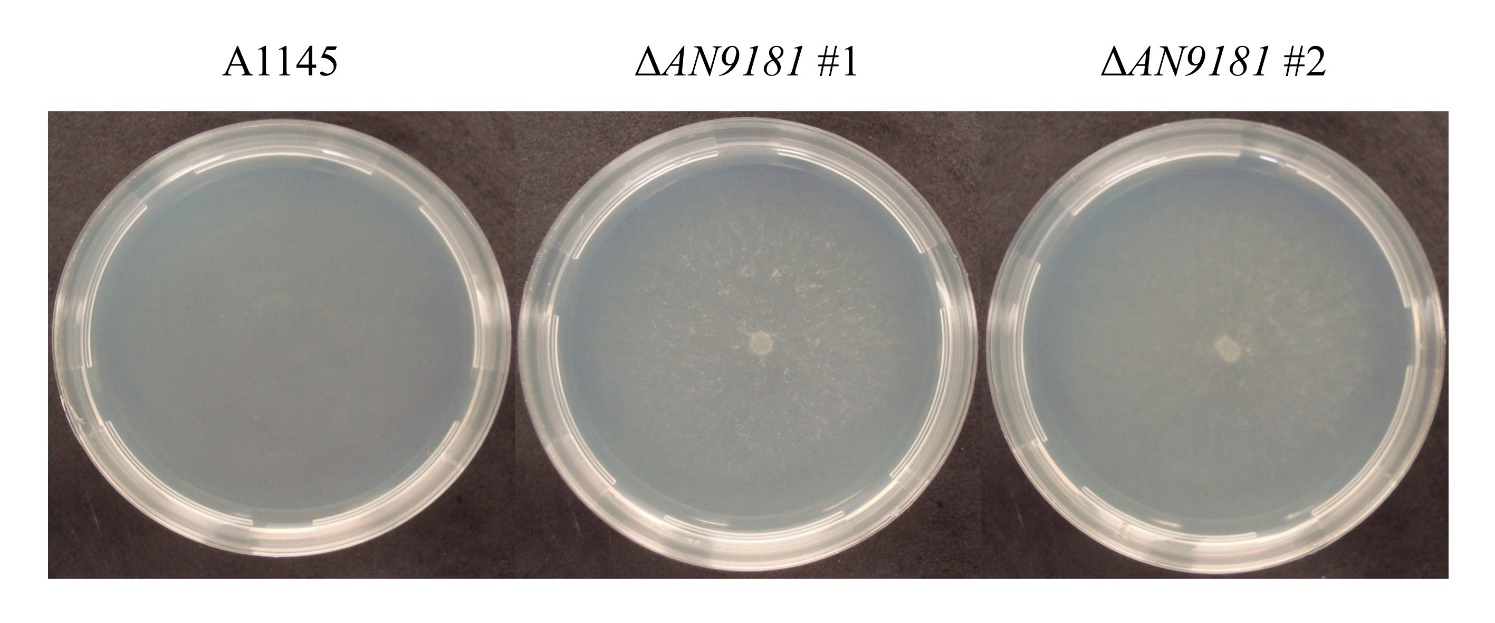


**Supplementary Figure 1:** **Confirmation of prototrophy for two colonies of A1145Δ*AN9181* deletion strain and auxotrophy for the parental strain A1145.** Growth on MMG without supplementation of uracil and uridine.


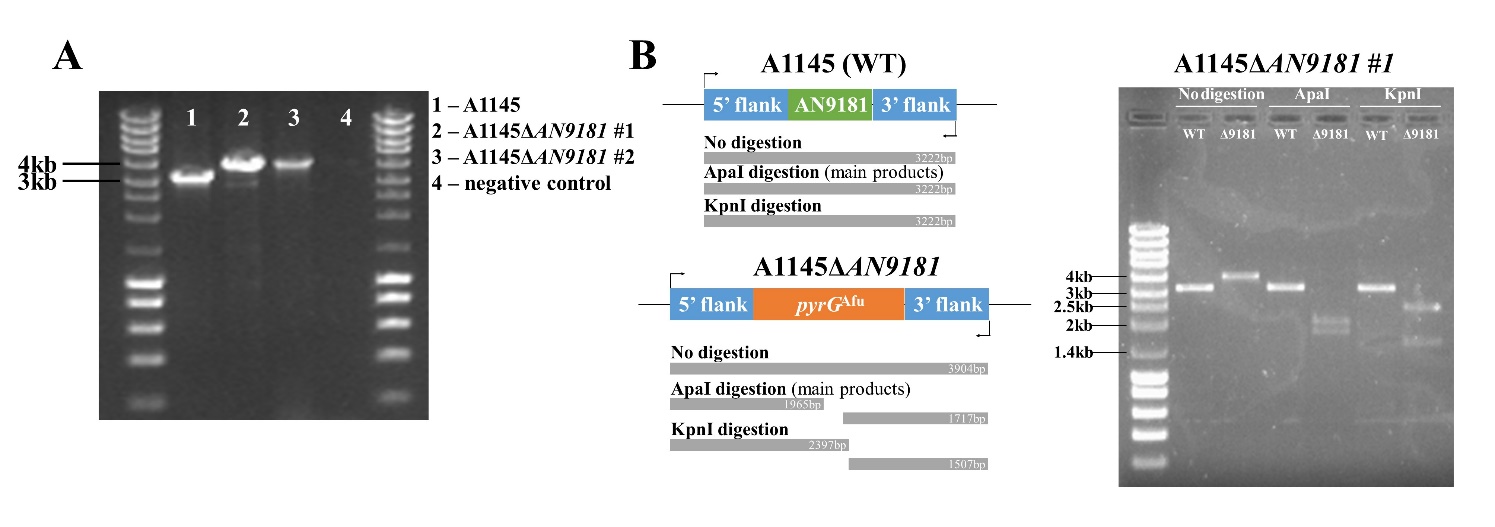


**Supplementary Figure 2:** **Confirmation of gene replacement A1145Δ*AN9181* strain.** Diagnostic PCR for parental strain and two colonies of the deletion mutant A1145Δ*AN9181* strain. (B) Digestion of the PCR product with the restriction enzymes ApaI or KpnI are represented for the wild-type strain and one of the colonies of the mutant. Gel electrophoresis (1% agarose) of the diagnostic PCR and digestion products confirms the correct gene replacement in the mutant strain.


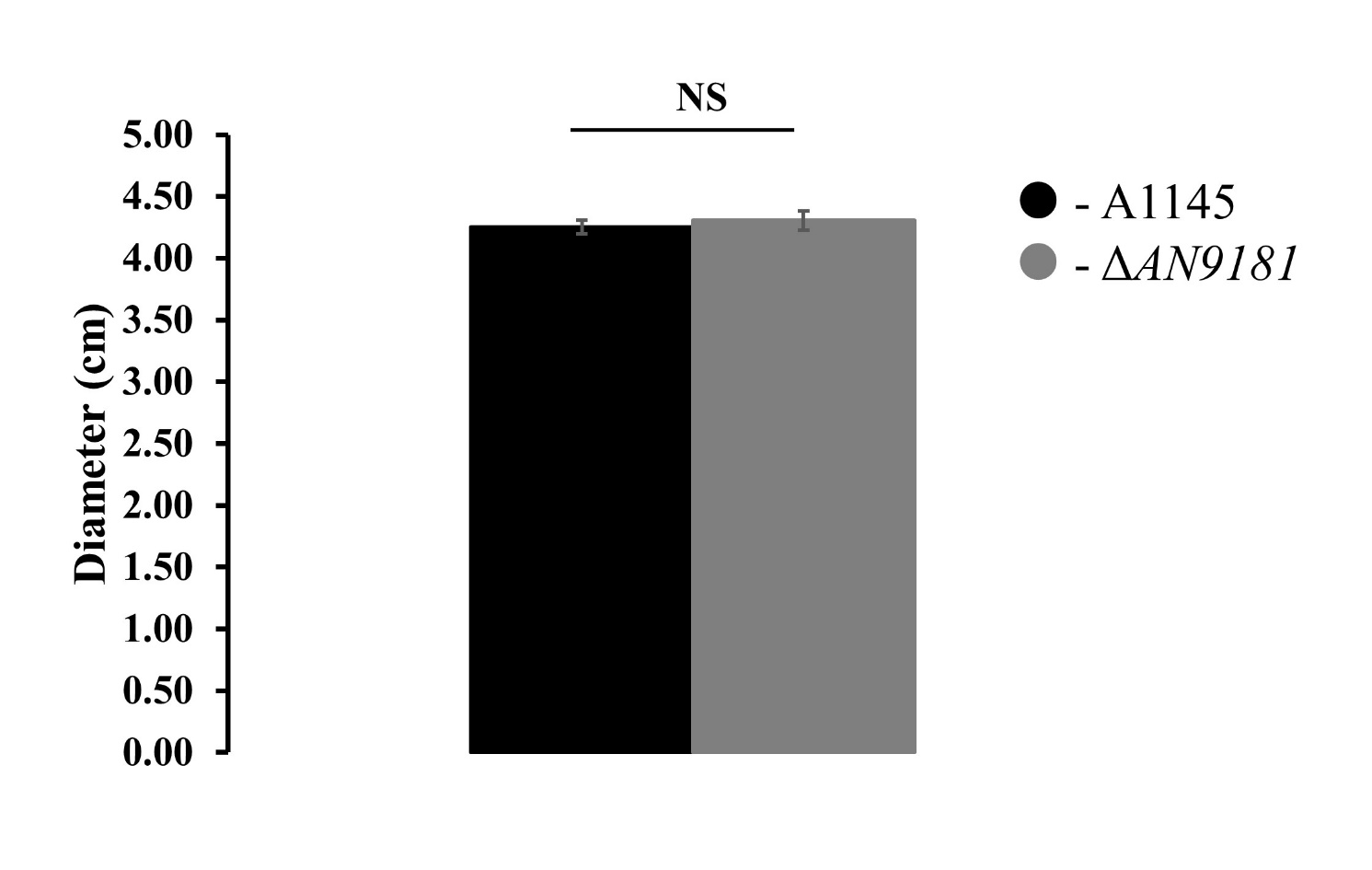


**Supplementary Figure 3:** **Phenotypic features of the deletion mutant strain as compared to the parental strain.** – Radial growth of Δ*AN9181* (gray) strain as compared to A1145 (black) parental strain grown on MMG agar plates.


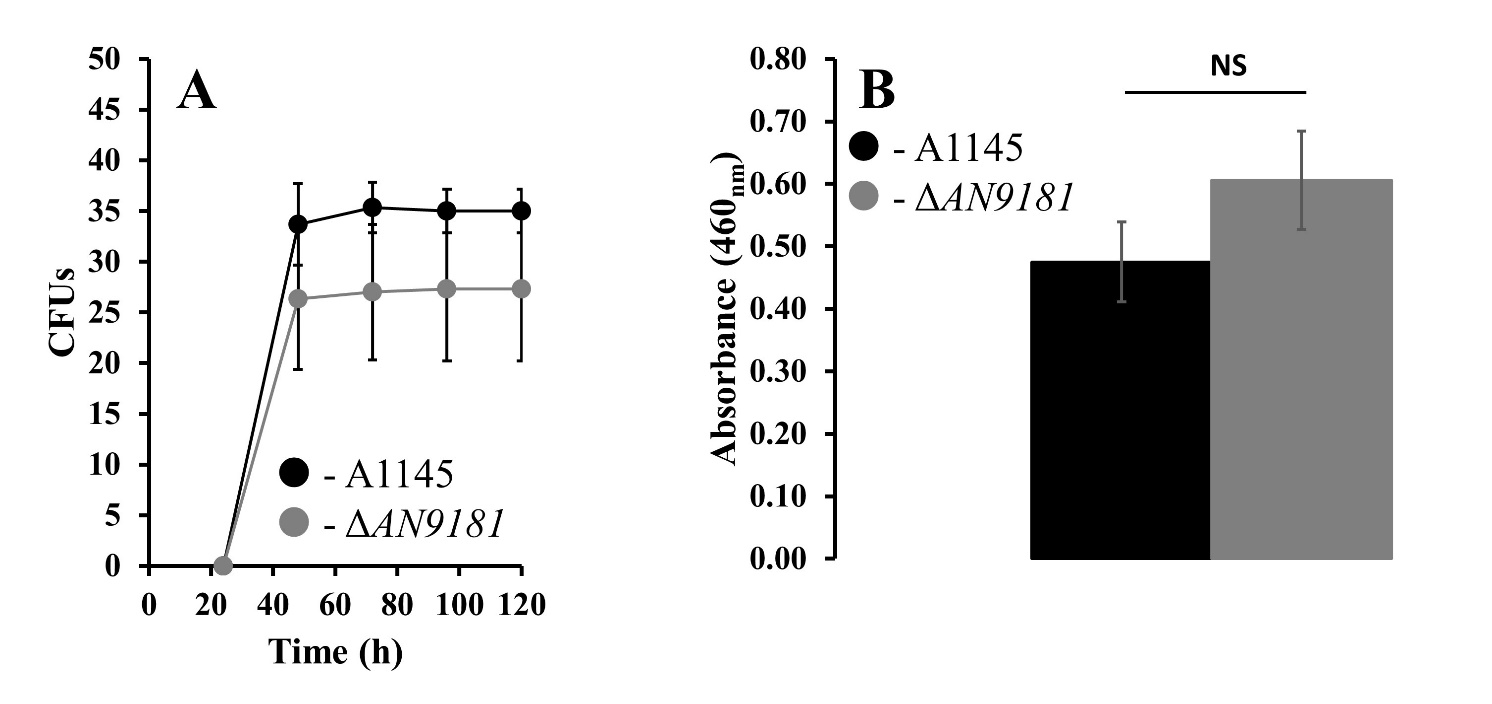


**Supplementary Figure 4:** **Phenotype features of A1145 and Δ*AN9181*.** (A) - Colony forming units of A1145 (black) and Δ*AN9181* (gray) plated on MMG supplemented with 71 mM nitrogen from NaNO_3_. (B) - Cell viability and proliferation measured by XTT assay. A1145 (black) and Δ*AN9181* (gray) strains were grown in MMG supplemented with 71 mM nitrogen from NaNO_3_. Values and error bars represent the mean and the standard deviation of triplicates. Significant differences (Student’s *t-test*) are marked with asterisks [*). * p ≤ 0.05; ** p ≤ 0.01 and *** p ≤ 0.001. “NS” means non-significant.


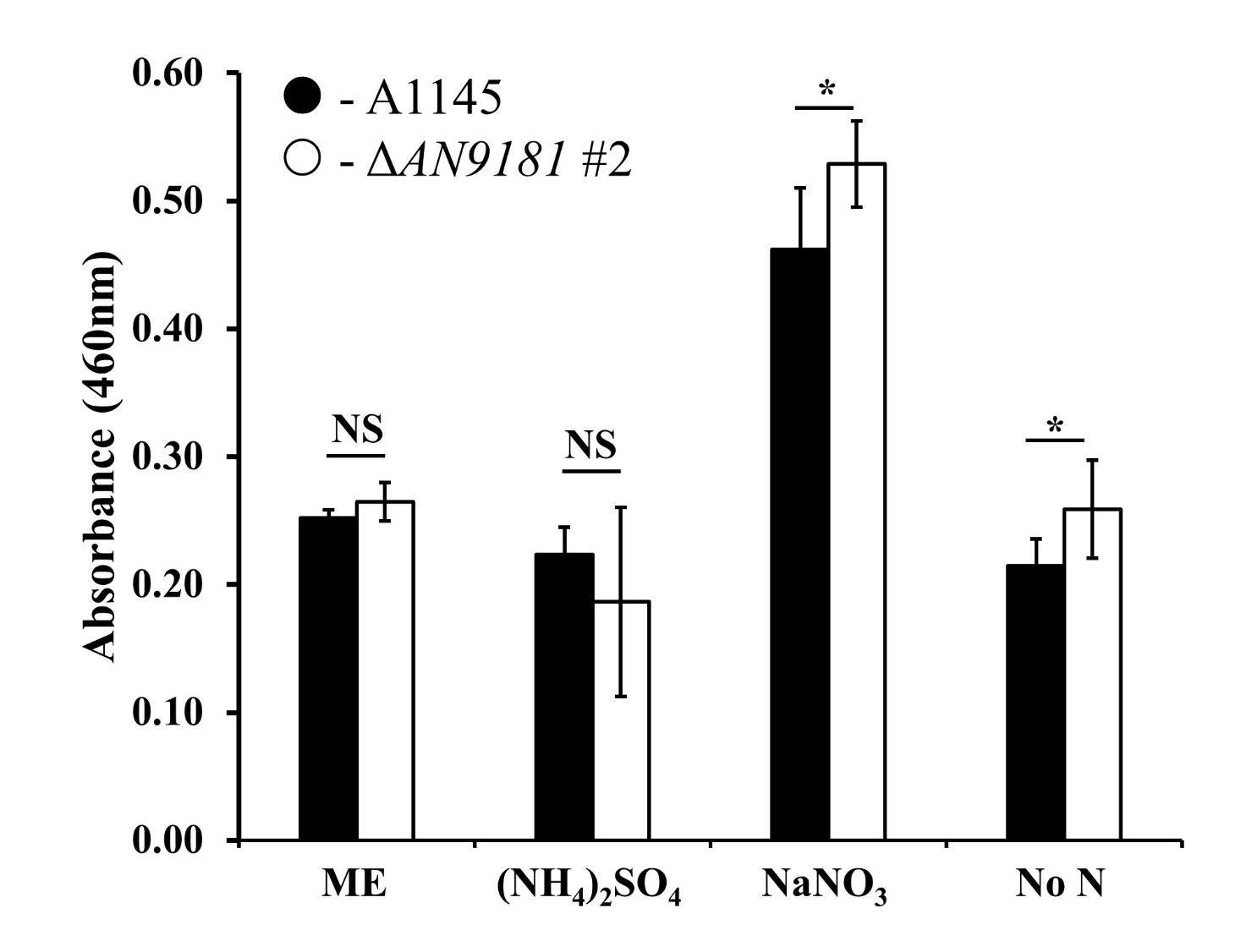


**Supplementary Figure 5:** **Cell viability and proliferation measured by XTT assay.** A1145 (black) and Δ*AN9181* #2 (white) strains were grown in Malt Extract or in minimal medium glucose supplemented with 10 mM nitrogen from the different nitrogen sources.


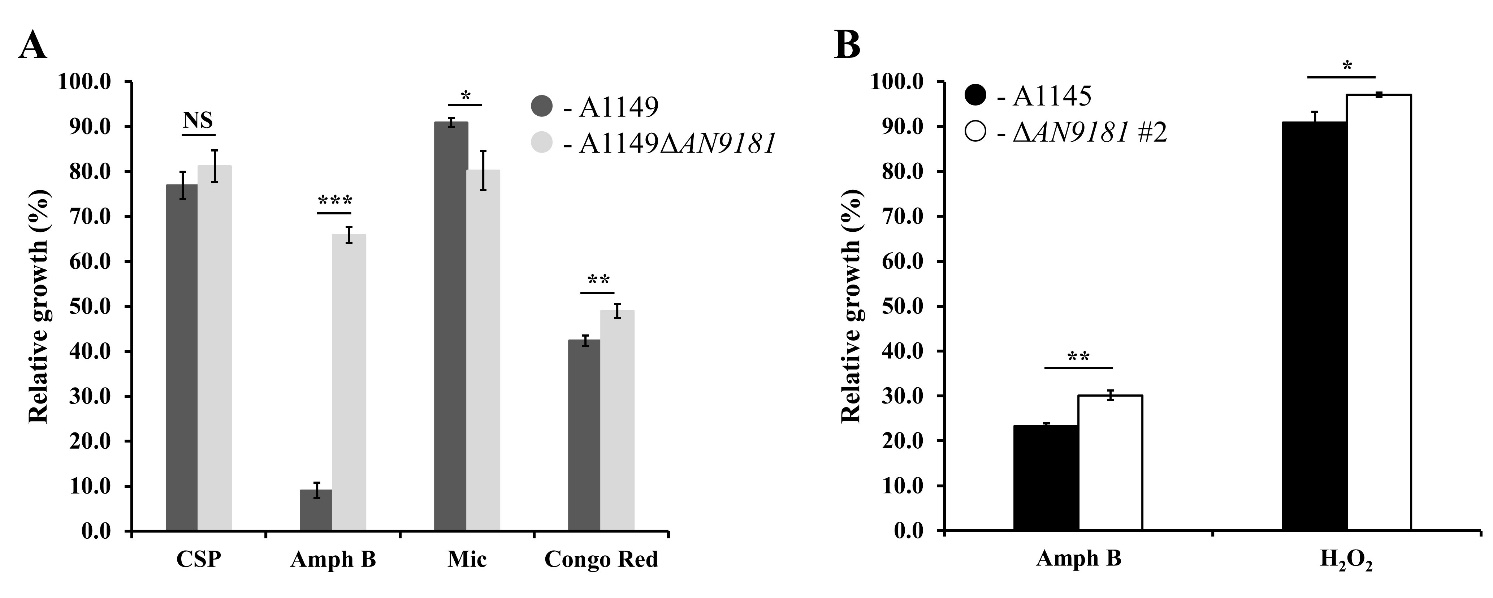


**Supplementary Figure 6:** **Phenotype features of A1149; A1149Δ*AN9181*; A1145 and A1145Δ*AN9181* #2.** – (A) relative growth of the A1149 (dark gray) and A1149Δ*AN9181* (light gray) strains grown in MMGN agar plates supplemented with: Caspofungin (0.15 mg·L^-1^), Amphotericin B (139 mg·L^-1^), Miconazole (0.5 mg·L^-1^) or Congo Red (0.05 mM) compared to the control condition (no chemical addition). (B) relative growth of the A1145 (black) and A1145Δ*AN9181* #2 (white) strains grown in MMGN agar plates supplemented with: Amphotericin B (150 mg·L^-1^) or H_2_O_2_ (2.5 mM) compared to the control condition (no chemical addition). Values and error bars represent the mean and the standard deviation of triplicate cultivations. Significant differences (Student’s *t-test*) are marked with asterisks [*). * p ≤ 0.05; ** p ≤ 0.01 and *** p ≤ 0.001. “NS” means non-significant.


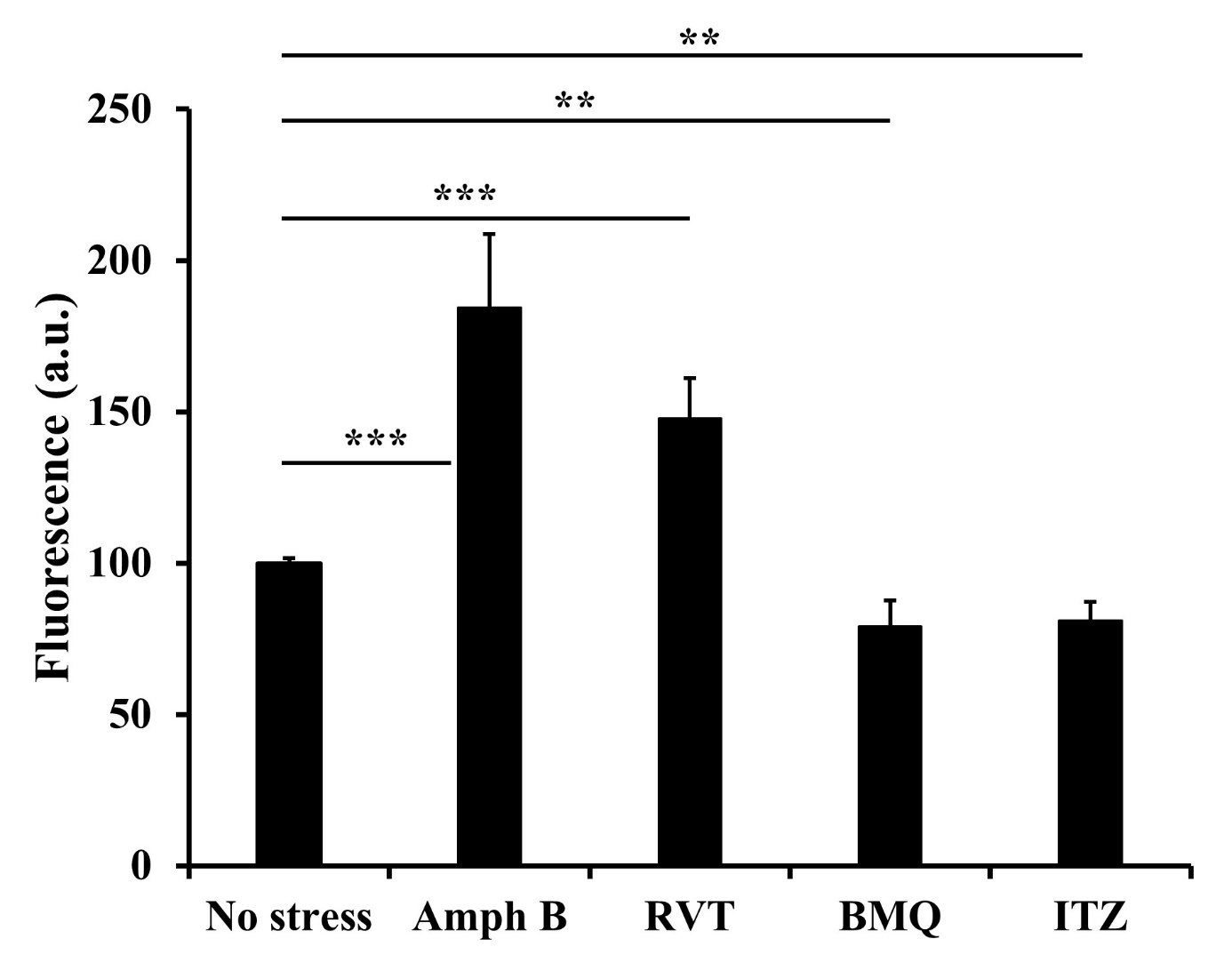
**Supplementary Figure 7:** **Intracellular ROS production.** The fluorescence intensity was measured for A1145 in the presence of Amph B; RVT; BMQ and ITZ. The fluorescence intensity per biomass dry weight amount of the control was defined as 100. Values and error bars represent the mean and the standard deviation of four replicates. Significant differences (Student’s *t-test*) are marked with asterisks [*). * p ≤ 0.05; ** p ≤ 0.01 and *** p ≤ 0.001. “NS” means non-significant.

## Supplementary Tables

**Supplementary table 1: List of oligonucleotide pairs used for RT-*q*PCR**

| **Gene name** | **Forward** | **Reverse** |
| --- | --- | --- |
| **AN3469** | CACCCGGACACTGGTATCTC | GAATACTTCGTAACGGCCTTGG |
| **AN8970** | CGGGTTATACGGGTGCATGA | CTCCAGCTGTTTCAGACCCC |
| **AN9181** | GATGTAGGGAAGAGGACCGC | ATTCGCACGACTCGGTTCTT |

**Supplementary table 2: The *Aspergillus nidulans* strains used in this study**

| **Strains** | **Genotype** | **Source** |
| --- | --- | --- |
| **A4** | wild type, *veA*+ | FGSC^a^ |
| **A1145** | *pyrG89*; *pyroA4*; *riboB*; *nkuA::argB* | FGSC^a^ |
| **A1145∆*AN9181*** | *pyrG*89; *pyroA*4; *riboB*; *nkuA*::*argB*; ∆*AN9181*::*pyrG^Afu^* | This study |
| **A1149** | *pyrG89*; *pyroA4*; *nkuA::argB* | FGSC^a^ |
| **A1149∆*AN9181*** | *pyrG89*; *pyroA4*; *nkuA::argB*; ∆*AN9181*::*pyrG^Afu^* | This study |

^a^ – Fungal Genetics Stock Center

**Supplementary table 3: Primers used in the generation of the gene-replacement mutants in *Aspergillus nidulans***

| **Gene** | **Primer** | **Sequence 5' – 3'** |
| --- | --- | --- |
| ***AN9181*** | AN9181_P1 | CTCCGAAGCGAGTCCGTC |
|  | AN9181_P2 | TGCTGCATACCTCACATGTTAG |
|  | AN9181_P3 | ATCCACTTAACGTTACTGAAATCCTGGGCAGGCCACGTAG |
|  | AN9181_P4 | GCTCCTTCAATATCATCTTCTGTCCGAGGGTGTAGTGAAGAAGTTG |
|  | AN9181_P5 | TTGAGATGCTGATCTCTGAGATC |
|  | AN9181_P6 | ACTTCTCCGCAGCTGCTG |
| ***pyrG^Afu^*** | CDS164 | GATTTCAGTAACGTTAAGTGGAT |
|  | CDS165 | GACAGAAGATGATATTGAAGGAGC |

**Supplementary table 4: PCR conditions used in the generation of gene-replacement mutants**

| **PCR** | **Reaction mix** | **PCR conditions** |
| --- | --- | --- |
| ***pyrG^Af^*** | 100 ng plasmid pCDS60 (for *pyrG^Afu^*)  0.3 µM primers CDS164/CDS165  0.4 mM dNTPs NZYmix  1.25 U Proof DNA polymerase  50 µl final volume | denaturation: 95 ºC, 3 min  35 cycles:  denaturation: 95 ºC, 30 s  annealing: 52 ºC, 30 s  extension: 72 ºC, 1 min/kb  final extension: 72 ºC, 5 min |
| **Flanking** | 100 ng *A*. *nidulans* A4 gDNA  0.3 µM primers P1/P3 or P4/P5  0.4 mM dNTPs NZYmix  1.25 U Proof DNA polymerase  50 µl final volume | denaturation: 95 ºC, 3 min  35 cycles:  denaturation: 95 ºC, 30 s  annealing: 52ºC, 30 s  extension: 72 ºC, 1 min/kb  final extension: 72 ºC, 7 min |
| **Fusion** | 150 ng *pyrG^Afu^* cassette  150 ng 5’-flank  150 ng 3'- flank  0.5 µM primers P2/P5  0.5 mM dNTPs NZYmix  5.0 U Long DNA polymerase  50 µl final volume | denaturation: 94 ºC, 2 min  35 cycles:  denaturation: 94 ºC, 20 s  annealing: 51 ºC, 30 s  extension: 68 ºC, 1 min/kb  final extension: 68 ºC, 10 min |
| **Diagnostic** | 2 µl transformant gDNA  0.5 µM primers P1/P6  0.5 mM dNTPs NZYmix  5.0 U Long DNA polymerase  50 µl final volume | denaturation: 94 ºC, 2 min  35 cycles:  denaturation: 94 ºC, 20 s  annealing: 60 ºC, 30 s  extension: 68 ºC, 1 min/kb  final extension: 68 ºC, 10 min |

**Supplementary table 5:** **EC_50s_ (mM)** for each of the organic compounds of the initial five transcriptomic datasets.

| **Organic compound** | ***A*. *nidulans* A4** |
| --- | --- |
| **Benzo[a]pyrene** | 1.61 |
| **5-Bromoquinoline** | 0.2 |
| **Sodium Salicylate** | 197.8 |
| **6-iodoquinoline** | 0.2 |
| **Resveratrol** | 0.45 |
| **Sodium Benzoate** | 54.8 |
